# Remotely Delivered Cognitive Behavioral Therapy for Adults With an Eating Disorder: Retrospective Analysis of a Real-World Patient Sample

**DOI:** 10.2196/76464

**Published:** 2025-09-18

**Authors:** Jessica H Baker, Nickolas M Jones, David Freestone, Lara Effland, Cara Bohon

**Affiliations:** 1 Equip Health, Inc Carlsbad, CA United States; 2 Psychiatry and Behavioral Sciences Stanford University Palo Alto, CA United States

**Keywords:** feeding and eating disorders, observational study, mental health, telemedicine, adult, cognitive behavioral therapy, outpatients, treatment outcome

## Abstract

**Background:**

Enhanced cognitive behavioral therapy (CBT-E) is the prevailing treatment approach for adult eating disorders. CBT-E is a variant of cognitive behavioral therapy, modified specifically to treat an eating disorder. Systematic reviews have established the effectiveness of CBT-E for adults when delivered face to face. However, few studies have evaluated evidence-based eating disorder treatment outcomes for programs intentionally designed to be delivered remotely.

**Objective:**

The objective of this study was to examine the clinical utility of CBT-E for adults with eating disorders using data from a national treatment program designed specifically for remote delivery.

**Methods:**

This was a pre-post observational cohort study conducted in a naturalistic setting where patients received treatment through standard clinical pathways, including typical referral, intake, and treatment processes. The participant sample for the study was identified through retrospective chart review and included adult patients (aged ≥18 y) diagnosed with anorexia nervosa, bulimia nervosa, binge eating disorder, or other specified feeding or eating disorder. For adult patients with these diagnoses, CBT-E is generally the first line of care in the program. CBT-E was developed to be transdiagnostic, and rather than focusing on a specific diagnosis, treatment focuses on treating the problematic beliefs related to weight, shape, and eating that maintain the eating disorder. CBT-E is highly individualized, and the treatment provider creates a treatment plan to match the specific eating disorder symptoms experienced by the patient. The recommended cadence of sessions is weekly. The criterion of utility was the magnitude and consistency of symptom change in weight gain and eating disorder, depression, and anxiety symptoms during CBT-E treatment. Survival analyses assessed patient and treatment characteristics. Multilevel models assessed the changes in outcomes both over time and at weeks 20 and 40, as these time points generally aligned with CBT-E clinical trial end points.

**Results:**

The patient sample (N=1629) predominantly consisted of White (n=1166, 71.6%), cisgender women (n=1403, 86.1%), with a mean age of 30 (SD 12) years. The overall median length of stay was 22 (95% CI 20-25) weeks. In all, 416 (25.5%) patients required weight restoration. The estimated probability of achieving weight restoration was 0.50 (95% CI 0.43-0.57) just before week 40 of treatment. By 40 weeks of treatment, the probability of achieving subclinical status for eating disorder symptoms was 0.48 (95% CI 0.44-0.51); for depression, the probability was 0.55 (95% CI 0.51-0.59), and for anxiety, the probability was 0.56 (95% CI 0.51-0.60). Time in treatment was significantly associated with improved symptoms across all outcomes (all *P*<.001).

**Conclusions:**

CBT-E delivered via telehealth is clinically useful, resulting in meaningful improvements in weight and eating disorder, depression, and anxiety symptoms in an outpatient setting. However, the absence of a comparison group and inclusion of a single treatment setting may limit generalizability.

## Introduction

### Eating Disorders

Eating disorders are behavioral conditions characterized by the severe and persistent disturbance in eating behaviors (eg, extreme food restriction, binge eating, and inappropriate compensatory behaviors for weight loss) associated with distressing thoughts and emotions [1]. Specific diagnoses may include anorexia nervosa (AN), bulimia nervosa (BN), and binge eating disorder (BED). The global lifetime prevalence of eating disorders is 3% to 5% [1], and the 1-year prevalence of eating disorders is comparable to other common mental health illnesses [2]. However, compared with conditions of a similar prevalence, access to treatment is worse [3]; approximately 80% of people with an eating disorder never receive care [4].

There are myriad structural and systemic reasons for the lack of access to care for people with eating disorders. Geographic location, long waiting lists for care, insurance coverage, high out-of-pocket costs, and stigma and shame associated with symptoms are just a few reasons contributing to access barriers [5-7]. Access to evidence-based care is particularly challenging given that such care is often limited to academic medical centers and requires extensive training by treatment providers [8]. These barriers result in an average time lag of 5 to 7 years between symptom onset and treatment [6,9].

### Cognitive Behavioral Therapy for Eating Disorders

Cognitive behavioral therapy (CBT) is the prevailing treatment approach for adult eating disorders [10-12]. Enhanced CBT (CBT-E) is a variant of CBT, modified specifically to treat adults with an eating disorder [13]. CBT-E was developed to be transdiagnostic, such that it involves targeting the maintaining mechanisms of an eating disorder rather than a specific diagnostic category. Systematic reviews and meta-analyses of CBT-E clinical trials have established the effectiveness of CBT-E for adults when delivered face to face [14-16].

While clinical trials have established the effectiveness of CBT-E for adult patients, they may have limited external validity. The treatment plan, patient sample, and criteria for success have been stringently specified a priori. Indeed, clinical trials have strict inclusion and exclusion criteria [17,18], and often exclude participants for certain comorbidities (eg, suicidality and substance use disorder) and psychosocial circumstances (eg, being unhoused and inability to commit to trial duration), the use of certain psychiatric medications, medical instability, and receiving previous treatment [15]. Although some studies suggest samples from clinical trials are representative, they still only represent people with the ability to access in-person care [19,20]. To bridge this gap, it is important to understand how eating disorder treatment delivered via CBT-E performs in the broader adult patient population.

### Remotely Delivered Treatment for Eating Disorders

Rates of remotely delivered treatments for mental health have risen significantly in recent years. Before 2021, less than 1% of mental health treatment visits occurred remotely, and it is now estimated that almost half of the mental health treatment visits occur remotely [21-23]. Given the increased availability, the desire for telemedicine by patients has also risen significantly in recent years [24]. Evidence-based eating disorder treatments developed for in-person care have demonstrated effectiveness for adults when delivered remotely [25-27]. For example, evaluating outcomes in an intensive outpatient program delivered in person and remotely showed no differences in outcomes based on delivery mode for eating disorder symptoms, symptoms of depression, and weight gain, and patients who began treatment face to face but were transitioned to remote delivery during the COVID-19 pandemic continued improving at the same rate as during face-to-face sessions [25-27]. The change from face-to-face to remote delivery of treatment was not associated with negative outcomes for 2 modalities of evidence-based care for an eating disorder (ie, CBT and family-based treatment) [28].

However, few studies have evaluated evidence-based eating disorder treatment outcomes for programs intentionally designed to be delivered remotely (ie, previous studies capitalized on an environmental event that required care to be delivered remotely), and studies examining the outcomes of adult patients receiving remotely delivered CBT-E are still limited. Given the barriers that exist to eating disorder treatment access, providing care via telehealth can make treatment available to adults who may not have been able to access care before.

### Objectives

The objective of this study was to examine the clinical utility of CBT-E for adults with eating disorders using data from a national treatment program designed specifically for remote delivery. The treatment program, originally implemented for youth and young adults aged between 6 and 24 years using family-based treatment, has demonstrated significant improvements in weight restoration and eating disorder symptoms [29]. Following the program’s expansion to serve adult populations, a naturalistic pre-post observational cohort study was conducted to evaluate changes in weight, eating disorder symptoms, and comorbid depression and anxiety over the course of remotely delivered CBT-E.

Given that the effectiveness of CBT-E has been well-established [14-16], our aim is not to compare CBT-E to other treatment modalities or no care but to examine if improvements in symptoms are achieved for patients receiving care. The referring criterion of utility is the magnitude and consistency of symptom change from admission to discharge across validated clinical measures. On the basis of the existing data and our previous findings for child and youth patients in our treatment setting [29], we hypothesized that our patients would have meaningful improvements in eating disorder–related symptoms during the course of treatment.

## Methods

### Treatment Program Overview

This study was conducted in a naturalistic setting in which patients received treatment through standard clinical pathways, including typical referral, intake, and treatment processes. Participants were drawn from an intentionally remote outpatient-level eating disorder treatment program within the US private health care system, which offers specialized care for those with an eating disorder. Potential patients voluntarily inquired about treatment through an online form or email. Inquiries were followed up by an admission specialist to determine appropriateness for outpatient eating disorder care. The US health care system is insurance based; therefore, before admission, insurance authorization and coverage were obtained for patients. However, patients were not required to have insurance to receive care and could cover costs out of pocket. Finally, potential patients deemed appropriate for care and interested in pursuing care were enrolled in the program and assigned to a care team. All patients were seeking care and self-selected into treatment. No recruitment was conducted, and treatment was voluntary and received as part of routine clinical care. Treatment was delivered remotely via a web-based platform.

### Participant Selection

The participant sample for the study was identified through retrospective chart review. Included patients were adults (aged ≥18 y) diagnosed with AN, BN, BED, or other specified feeding or eating disorder (OSFED) who began treatment between January 1, 2023, and December 31, 2024. Eating disorder diagnosis was based on a semistructured clinical interview by at least a master’s level mental health therapist during a comprehensive intake appointment. Patients included those who were discharged or currently receiving care. This resulted in a patient sample of 1629.

### Ethical Considerations

The evaluation of the program’s patient treatment outcomes was reviewed by the Western institutional review board (20216235). It was determined that the evaluation of patient treatment outcomes did not meet the definition of human participant research and was therefore considered exempt from institutional review board oversight. Patients also provided consent to treatment information being used for research purposes as part of the admissions process. Data used for analysis were obtained from electronic medical records. The analytic sample was deidentified and included only the minimal amount of information necessary to complete the objective. All data collection and storage procedures were Health Insurance Portability and Accountability Act (HIPAA)-compliant.

### Treatment Overview

All patients were assigned to a multidisciplinary care team that included a therapist, dietitian, peer mentor, and medical provider as needed; patients also had the option to include a psychiatrist. The peer mentor had recovered from an eating disorder and provided a source of hope and motivation for the patient to see that recovery is possible. Patients were also encouraged to have a support person as part of treatment. A support person is anyone (eg, family, friend, relative, or another person with a close relationship to the patient) who is relationally appropriate and able to join the patient in treatment. Identified supporters had access to a family mentor. A family mentor is someone who has helped support a loved one through recovery and aids the patient’s support person through the treatment process. All care was delivered through an internally developed, HIPAA-compliant web-based platform. Treatment was voluntary, and the end of treatment was based on clinical progress.

For adult patients diagnosed with AN, BN, BED, and OSFED, CBT-E is generally the first line of care in the program. The treatment structure is adapted from *Cognitive Behavior Therapy and Eating Disorders* [13] and *Cognitive Behavior Therapy for Adolescents with Eating Disorders* [30]. CBT-E was developed to be transdiagnostic. Rather than focusing on a specific diagnosis, CBT-E focuses on treating the problematic beliefs related to weight, shape, and eating that are maintaining factors of the eating disorder. The maintaining factors are present on a spectrum of behaviors for the specific patients in care; as the disorder evolves over time, CBT-E is highly individualized, and the therapist creates a CBT-E treatment plan to match the eating disorder symptoms experienced.

### CBT-E Treatment Overview

The cognitive behavioral theory that underpins CBT-E focuses on the current maintaining mechanisms of the disorder rather than those responsible for its initial development [13]. CBT-E guides a patient through the stages of change by first identifying the current maintaining mechanisms and then implementing effective interventions and skills. CBT-E has 4 stages [13]. Stage 1 focuses on engaging the patient in the treatment and building motivation for change through rapport and trust with the team, psychoeducation about the disorder, case formulation, and the pros and cons of recovery. Stage 2 is a bridge stage for the treatment team to review the patient’s progress in stage 1 and identify areas of improvement or issues with commitment. Stage 3 is the core of CBT-E, where the maintaining factors of a disorder specific to each patient are targeted. During stage 3, each patient works through 1 of the 5 modules (ie, eating and weight restoration; body image; restraint and restriction; mindset and setbacks; and events, moods, and eating). Each module includes CBT skills and interventions to teach the patient a new effective response to the maintaining factors of the eating disorder. Stage 3 is the longest portion of treatment and can take 10 to 30 weeks to complete, depending on the severity of the behaviors and negative impact on the patient. Stage 4 is the final stage and focuses on planning for the future and relapse prevention. The recommended cadence of sessions is weekly with a dietitian or a therapist until the patient significantly reduces behaviors maintaining the eating disorder. Dietitians take the lead on technical aspects of weight restoration and dietary rule challenges, and therapists focus on the psychological mechanisms maintaining the disorder, emotional regulation, and motivation. This collaborative approach ensures patients receive comprehensive care while allowing each provider to work within their area of expertise.

Finally, the CBT-E program was modified slightly for remote care and inclusivity. Specifically, language was modified for weight inclusivity such that weight was masked unless deemed clinically appropriate. Broad modules were offered via groups to ease the training burden and aid in implementation of additional evidence-based treatment strategies that target core psychological barriers to eating disorder recovery (eg, perfectionism, core low self-esteem, and emotion regulation skills). Licensed therapists received approximately 10 hours of training and 12 hours of consultation after training. Therapists and dietitians continued with clinical supervision and consultation via weekly individual and group supervision.

### Study Design and Overview

This was a pre-post, within-subject observational cohort study. We evaluated the clinical utility of remotely delivered CBT-E by examining the magnitude and consistency of symptom change from admission to discharge across validated clinical measures, as described subsequently.

#### Measures

To evaluate treatment outcomes, we examined various outcomes throughout the treatment. Measures were completed as part of standard care on the HIPAA-compliant treatment platform.

#### Weight

Weight was collected using BodyTrace Scale, a connected device that automatically sends weight data electronically to the electronic medical record, starting in March 2024. Before this, patients completed weight data collection via in-person appointments at primary care or student health centers, or self-reported weight using a scale at home. Patients were instructed to measure weight twice weekly with minimal clothing, after voiding, and before food or beverage consumption. For patients needing weight restoration, the target weight was determined by the registered dietitian using the Centers for Disease Control and Prevention [31] age-adjusted BMI growth charts and the patient’s individual growth trajectory from historical medical records [32]. Weight restoration was defined as achieving 95% of the target weight set by a practitioner at the onset of treatment. Analyses evaluating weight change only included patients with a weight restoration treatment goal***.***

#### Eating Disorder Symptoms

To assess eating disorder symptoms, patients completed the Eating Disorder Examination Questionnaire (EDE-Q) [33] monthly for the first 3 months of treatment and quarterly thereafter. The EDE-Q has 28 items and is made up of 4 subscales (restraint, eating concerns, weight concerns, and shape concerns) and a global score. A global score of 2.8 or more suggests clinically significant eating disorder symptoms [34]. Internal consistency was excellent (Cronbach α=0.92).

#### Depression

Patients completed the Patient Health Questionnaire-8 (PHQ-8) [35,36] monthly for the first 3 months of treatment and quarterly thereafter. PHQ-8 reflects the *DSM* (*Diagnostic and Statistical Manual of Mental Disorders*) diagnostic criteria for depression and is a valid measure for depression across diverse populations [37]. PHQ-8 asks about the frequency of depressive symptoms within the past 2 weeks. Response options range from 0 (not at all) to 3 (nearly every day). The numeric response scores for each item are summed together to create a total score. Higher scores indicate more severe depressive symptoms. A score of 10 or more is considered the clinical cutoff, indicating the need for further evaluation [35,36]. The PHQ-8 had good internal consistency in this sample (Cronbach α=0.85).

#### Anxiety

Patients completed the Generalized Anxiety Disorder-7 (GAD-7) [38] questionnaire monthly for the first 3 months of treatment and quarterly thereafter. The GAD-7 reflects diagnostic criteria for generalized anxiety disorder. The GAD-7 asks about the frequency of anxiety symptoms within the past 2 weeks. Response options range from 0 (not at all) to 3 (nearly every day). Ratings are summed such that total scores can range from 0 to 21, where higher scores indicate more severe anxiety symptoms. A score of 10 or more is considered the clinical cutoff, indicating the need for further evaluation [38]. The GAD-7 had good internal consistency in this sample (Cronbach α=0.89).

### Analytic Strategy

#### Patient and Treatment Descriptions

Descriptive analyses were conducted to characterize the patient sample. We report sample sizes (counts and percentages) where appropriate. Survival analyses were used to determine (1) the median length of stay in treatment; (2) the proportion of patients meeting weight restoration targets; and (3) the proportion of patients who achieved subclinical status on eating disorder, anxiety, and depression symptom scales over treatment time. Survival models result in inferential time-to-event estimates and estimated survival probabilities over time (there are no counts to report).

#### Change in Outcomes Over the Course of Treatment

Patients were asked to complete surveys and input weight measurements multiple times throughout treatment; outcome measurements over time were nested within patients. Unlike clinical trials, which typically have a specified treatment end point, treatment length among patients in this real-world sample was variable. Thus, we modeled outcomes through 1 year of treatment. Using these models, we estimated treatment outcomes at weeks 20 and 40, as these time points generally align with CBT-E clinical trial end points [39,40] as well as the 1-year time point (week 52 of treatment).

To provide accurate estimates of these outcomes at specific weeks of treatment, we fit outcome trajectories using a series of multilevel models, one for each measurement type (weight, EDE-Q, PHQ-8, and GAD-7). Each model took the following form:

outcome_w,j_ ~ β_0_+ β_0j_ + β_1_log(w) + β_1j_log(w) + β_2_age + β_3_[age × log(w)] + β_4_gender + β_5_[gender × log(w)] + β_6_diagnosis + β_7_[diagnosis × log(w)]

where *w* represents the treatment week (we took the log because treatment progresses logarithmically over time; [29,41]), and *j* indexes the patient (so that β_0j_ and β_1j_ represent random intercepts and slopes). The random effects accounted for different patient-level starting points and outcome trajectories over time. We reported unstandardized results for the full sample and by patient diagnosis. However, the aim of this paper was not to compare the effectiveness of CBT-E across eating disorder diagnoses; hence, results are shown by diagnosis for descriptive purposes only.

All analyses were performed in R software (version 4.4.2; R Foundation for Statistical Computing) using the *tidyverse* package (version 2.0.0). Survival analyses were performed using the packages *survival* and *survminer* (version 0.5.0); multilevel modeling was performed using *lme4* (version 1.1-35.5), *ggeffects* (version 2.0.0), and *emmeans* (version 1.10.6). Project data management and workflow were managed using DuckDB (version 1.1.3-1) and targets (version 1.9.1).

#### Missingness

We used all available patient data and did not exclude patients based on data completeness; therefore, different analyses may have slightly different analytic sample sizes. Some patients completed more survey responses than others, and some patients were missing information necessary for 1 analysis but not for others. In addition, we began administering the EDE-Q survey in August 2023; therefore, a small number (approximately 9%) of the patients were already in or had completed treatment by August 2023. Thus, EDE-Q scores were missing for these patients before August 2023. Patients were more likely to complete outcome surveys (ie, EDE-Q, GAD-7, and PHQ-8) early in treatment compared to later (all *P*s<.001).

Older adult patients were slightly more likely to fill out the surveys compared with younger adults (all *P*s<.001). Survey completion for these outcomes was not related to any other variable used in this study, including gender, diagnosis, and symptom severity as indicated by scores over treatment time. Older adults were more likely to provide weight measurements (*P*<.001); however, missingness on weight was not related to a patient’s overall weight during treatment. We report complete-case analysis, but using imputation methods (via the *mice* package in R) gave the same pattern of results and nearly identical estimated coefficients.

## Results

### Patient and Treatment Characteristics

Patient demographics are presented in Table 1 (N=1629). The patient sample was predominantly composed of cisgender women (n=1403, 86.1%) and white individuals (n=1166, 71.6%). The average patient age was 30 (SD 12.00) years. In total, 913 (56%) patients were diagnosed with AN, 388 (23.8%) with BED, 120 (7.4%) with BN, and 208 (12.8%) with OSFED. Nearly one-third (n=487, 29.9%) of the patients self-reported receiving treatment at a higher level of care facility (eg, residential) at some point before the current treatment. Patient characteristics by diagnosis are presented in Table S1 in Multimedia Appendix 1.

**Table 1:** Patient sample characteristics (N=1629).

Characteristic			Values
Age (y), mean (SD)			30 (12.00)
**Gender, n (%)**			
	Cisgender woman	1403 (86.1)	
	Cisgender man	128 (7.9)	
	Transgender or nonbinary	86 (5.3)	
	Missing gender	12 (0.7)	
**Race and ethnicity, n (%)**			
	Asian	84 (5.2)	
	Black or African American	66 (4.1)	
	Hispanic	94 (5.8)	
	White	1166 (71.6)	
	Multiethnic or multiracial	166 (10.2)	
	Other	28 (1.7)	
	Missing race and ethnicity	25 (1.5)	
**Diagnosis, n (%)**			
	AN^a^	913 (56)	
	BED^b^	388 (23.8)	
	OSFED^c^	208 (12.8)	
	BN^d^	120 (7.4)	
**Previous treatment experience, n (%)**			
	No previous treatment	876 (53.8)	
	Previous HLOC^e^	487 (29.9)	
	Unknown	199 (12.2)	
	Other previous treatment	67 (4.1)	
**Comorbidities, n (%)**			
	Depression	966 (59.3)	
	Anxiety	730 (44.8)	
	ADHD^f^	381 (23.4)	
	PTSD^g^	365 (22.4)	
	OCD^h^	287 (17.6)	
	None	261 (16)	
	Substance use	51 (3.1)	

^a^AN: anorexia nervosa.

^b^BED: binge eating disorder.

^c^OSFED: other specified feeding or eating disorder.

^d^BN: bulimia nervosa.

^e^HLOC: higher level of care.

^f^ADHD: attention deficit/hyperactivity disorder.

^g^PTSD: posttraumatic stress disorder.

^h^OCD: obsessive-compulsive disorder.

At intake, patients self-reported previous psychological diagnoses. Of the 1629 patients, 966 (59.3%) self-reported major depressive disorder, 730 (44.8%) self-reported generalized anxiety disorder, 381 (23.4%) self-reported attention-deficit/hyperactivity disorder, 365 (22.4%) self-reported posttraumatic stress disorder, and 287 (17.6%) self-reported obsessive-compulsive disorder. In total, 261 (16%) self-reported no comorbid diagnoses.

Of the 1629 patients, 416 (25.5%) required weight restoration. The median weight gain needed to reach the target weight was 7.35 kg (16.2 lbs). BMI at admission for patients on weight restoration ranged from 12.07 to 35.93, with a median of 18.93; BMI at the 75th percentile was 20.78, indicating that 75% (298/397) patients had a BMI of 20.78 or less. Table S2 in Multimedia Appendix 1 presents BMI by diagnosis.

In total, 82.6% (880/1065; with an initial EDE-Q score) of the patients entered treatment with clinically significant eating disorder symptoms (EDE-Q score of >2.8), 62.5% (723/1157; with a starting PHQ-8 score) of the patients entered treatment with clinically significant depression symptoms (PHQ-8 score of >10); and 55.8% (647/1159; with a starting GAD-7 score) of the patients entered treatment with clinically significant anxiety symptoms (GAD-7 score of >10).

The overall median length of stay was 22 (CI 20-25) weeks. Length of stay was significantly longer for patients with OSFED (41, CI 32-51 weeks) relative to all other diagnoses. In treatment, patients were primarily engaged with therapists and dietitians; other, less frequent sessions included meeting with a physician, a psychiatrist (if needed), and peer and family mentors. Patients attended an average of 22.9 (SD 21.6) therapy sessions throughout treatment, a rate of approximately 0.73 sessions per week, and they attended an average of 16.8 (SD 16.2) dietitian sessions, a rate of approximately 0.57 sessions per week. At the onset of treatment, the median number of weekly sessions was approximately 2 (range 1-6; usually a therapist appointment, dietitian appointment, and one other type of appointment); the median number of weekly sessions tapered over time down to roughly 1 session per week (range 1-5) by week 40 of treatment.

### Change in Outcomes Over the Course of the Treatment

**Figure 1:**
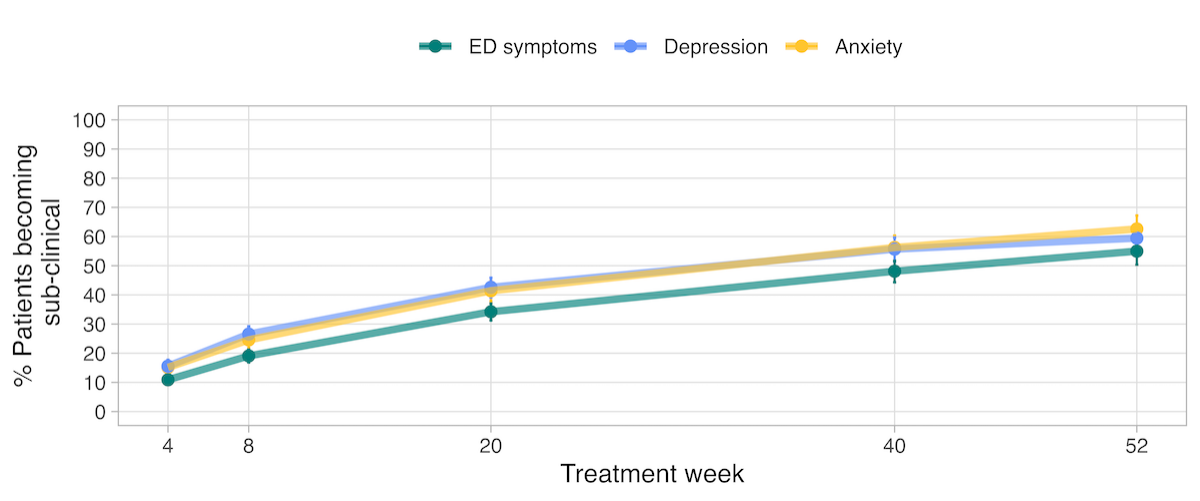
The percentage of patients who achieved subclinical status for eating disorder (ED) symptoms, depression, and anxiety across treatment time. Subclinical levels for anxiety are indicated by a score of <10 on the Generalized Anxiety Disorder-7, a Patient Health Questionnaire-8 score of <10 for depression, and an Eating Disorder Examination Questionnaire score of <2.8 for eating disorder symptoms.

In a survival analysis of 321 patients on weight restoration with set target weights, we found that the estimated probability of achieving weight restoration (95% of target weight) was 0.50 (CI 0.43-0.57) just before week 40 of treatment. By week 52, the probability was 0.53 (CI 0.45-0.60). Survival analyses of time to achieve subclinical status on EDE-Q, PHQ-8, and GAD-7 (Figure 1) showed that by 20 weeks of treatment, the probability of achieving subclinical status on EDE-Q was 0.34 (CI 0.31-0.37); for depression, the probability was 0.42 (CI 0.39-0.45), and for anxiety, the probability was 0.41 (CI 0.37-0.44). By 40 weeks of treatment, the probability of achieving subclinical status on EDE-Q was 0.48 (CI 0.44-0.51); for depression, the probability was 0.55 (CI 0.51-0.59), and for anxiety, the probability was 0.56 (CI 0.51-0.60). By 52 weeks of treatment, the probability of achieving subclinical status on EDE-Q was 0.54 (CI 0.50-0.59); for depression, the probability was 0.59 (CI 0.54-0.63), and for anxiety, the probability was 0.62 (CI 0.57-0.67).

**Figure 2:**
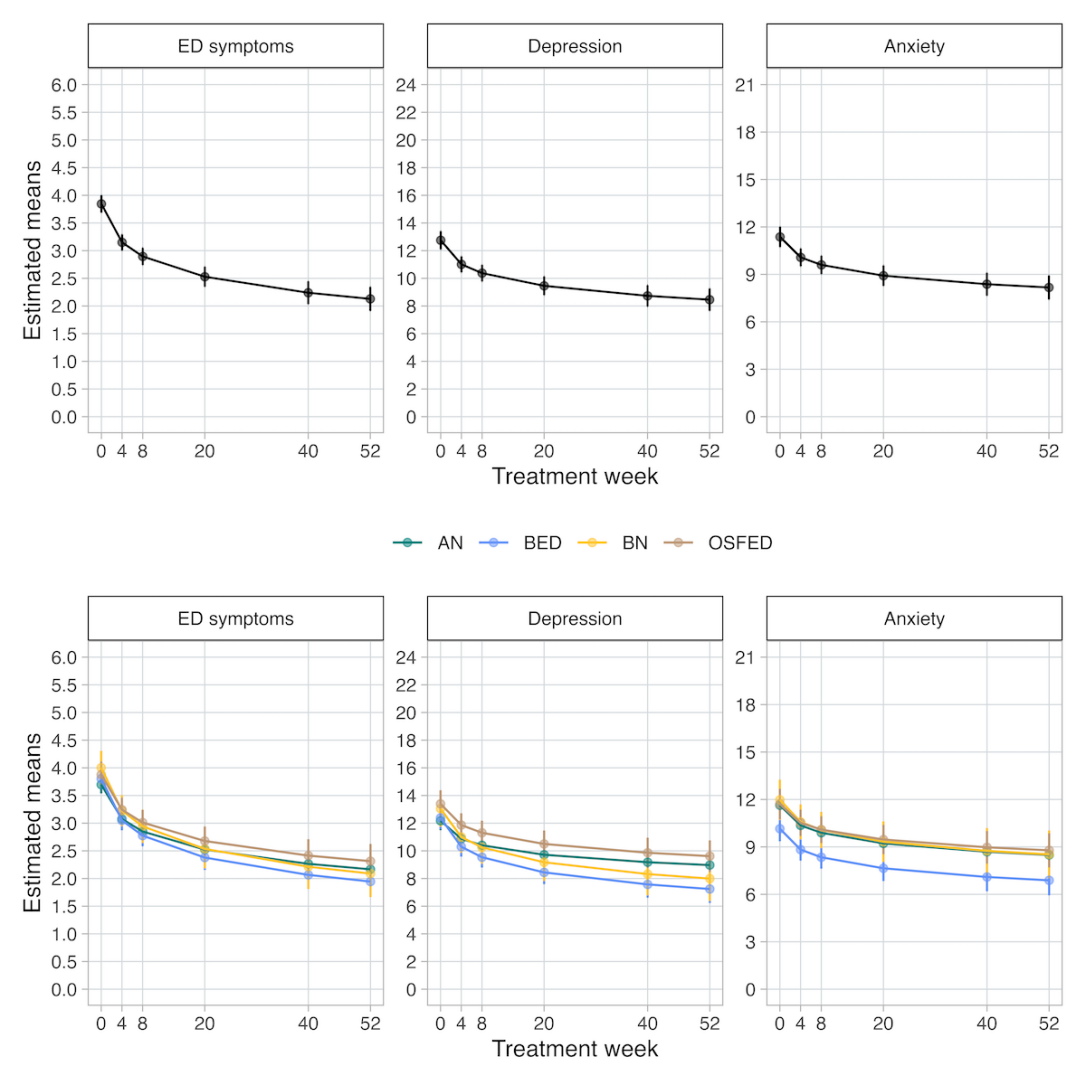
Model-derived estimates of outcomes over treatment time for the overall sample and by diagnosis. AN: anorexia nervosa, BED: binge eating disorder; BN: bulimia nervosa; ED: eating disorder; OSFED: other specified feeding or eating disorder.

Multilevel model results for each outcome are presented in Table S3 in Multimedia Appendix 1. Treatment time (wk) was significantly associated with improved symptoms across all outcomes (EDE-Q: b=–0.40, SE 0.02; *P*<.001; PHQ-8: b=0.86, SE 0.09; *P*<.001; GAD-7: b=–0.87, SE 0.09; *P*<.001). Overall, mean EDE-Q scores decreased from 3.84 (95% CI 3.69-4.00) to 2.53 (95% CI 2.35-2.71) at week 20 and 2.24 (95% CI 2.03-2.44) at week 40; mean PHQ-8 scores decreased from 12.8 (95% CI 12.09-13.41) to 9.46 (95% CI 8.78-10.13) at week 20 and 8.73 (95% CI 7.97-9.50) at week 40; mean GAD-7 scores decreased from 11.40 (95% CI 10.72-12.01) to 8.92 (95% CI 8.27-9.56) at week 20 and 8.38 (95% CI 7.66-9.10) at week 40. Moreover, symptom improvement was not significantly moderated by age or gender. In general, symptoms improved similarly across diagnoses (Figure 2; underlying estimates by diagnosis are available in Table S4 in Multimedia Appendix 1 along with week 52 estimates).

## Discussion

### Overview

In a naturalistic evaluation of the clinical utility of remotely delivered CBT-E for adults with an eating disorder, the treatment resulted in meaningful clinical improvements. Patients who received CBT-E in the intentionally remote treatment program for up to 1 year showed significant improvements in weight restoration, eating disorder symptoms, and comorbid depression and anxiety.

### Principal Findings

CBT-E delivered remotely in an outpatient setting by a multidisciplinary team produced clinically meaningful improvements across eating disorder diagnoses. By week 40, the estimated probability of being weight restored was 0.50. Similarly, for patients who initially reported clinically significant ED, depression, or anxiety scores, the probability of reaching subclinical levels by week 40 was 0.48, 0.55, and 0.56, respectively. On average, symptom score averages also decreased from clinically significant at admission to subclinical levels by week 20.

The goal of this study was not to compare CBT-E clinical improvements among eating disorder diagnoses; however, we report symptom outcomes by diagnosis for informational purposes. A similar pattern was observed across all diagnoses for all symptoms, such that self-report symptom scores continually and similarly decreased (ie, symptom improvement) across time in treatment. Taken together, our results provide preliminary evidence that CBT-E delivered remotely is a reasonable treatment approach for transdiagnostic eating disorders in an outpatient setting.

### Comparison to In-Person Studies

The findings reported in this study generally align with clinical trials [39,40] evaluating the effectiveness of CBT-E for adults delivered in person. In general, in-person treatment studies show that approximately 50% of patients have a BMI greater than 18.5 by the end of the treatment (for those that need to gain weight) [42-45]. Similarly, in this study, we found that the probability of being weight restored by week 40 of treatment was 0.50. In other words, we would expect that approximately 50% of patients in treatment would achieve the target weight assigned to them by their treatment team by that point in treatment. However, for some in-person clinical trials, the weight outcome of 18.5 or more [46-48] does not mean the patient achieved their target weight; it simply means that the weight was no longer in an underweight BMI category. A patient could need several more weeks or months of treatment to achieve their target weight. These different definitions of weight restoration make direct comparisons between our patients’ weight outcomes to those reported in some in-person clinical trials difficult.

In-person clinical trials also indicate that approximately 30% to 60% of the patients with AN or BN are defined as in remission (definitions may vary) [42-46]. Although we did not directly evaluate remission or recovery status in our patient sample (given the lack of a predefined treatment end point and that patients could still be actively engaged in treatment), our results still align with this finding. By week 40 of treatment, the probability of reaching subclinical levels of eating disorder, depression, and anxiety symptoms were 0.48, 0.55, and 0.56, respectively. Thus, we would expect that approximately 48% of patients with clinical ED symptoms at treatment onset would improve to subclinical levels after 40 weeks of treatment, whereas 55-56% of patients who had clinical levels of depression or anxiety at treatment onset would improve to subclinical levels by 40 weeks of treatment. Taken together, our findings preliminarily suggest that CBT-E delivered remotely within our treatment program results in similar improvements to in-person trials.

### Translating Clinical Trials to Real-World Care

Although it is not the primary aim of this study, our findings show the complexities of translating results from controlled studies to a general patient population. For example, clinical trials of CBT-E typically have a fixed end point for treatment at approximately 20 or 40 weeks (depending on weight status). However, in our transdiagnostic sample of patients, the overall median length of treatment was 22 (IQR 8-52) weeks. Given the variability of real-world treatment, it is challenging to make comparisons with clinical trial end-of-treatment outcomes.

Furthermore, clinical trials have strict criteria for study inclusion, which may limit the generalizability of the results to the typical patient [17,18]. For patients with eating disorders, comorbidity is the rule rather than the exception [49], and clinical trials often exclude patients with certain comorbidities that are common in eating disorders (eg, suicidality, substance use disorder, and medical instability). The ability to access and join a clinical trial is also limited. Given these added challenges, the typical patient with an eating disorder may have additional barriers to success compared with those enrolled in a clinical trial. Even trials designed to be “inclusive” may not represent the average patient with an eating disorder (eg, inclusion and exclusion criteria may be stricter, including BMI criteria, previous similar treatment requirements, and consent to research) [42]. Therefore, it is also important to consider patient outcomes within the context of such additional confounding factors that may have an impact on the length of care and treatment progress.

### Strengths and Limitations

This study has several strengths. This is the largest study to date evaluating the clinical utility of CBT-E for transdiagnostic eating disorders delivered in a remote outpatient clinic setting by a multidisciplinary team. We used rigorous modeling techniques to derive estimates of treatment outcomes in this setting to show the clinical utility of CBT-E in this transdiagnostic sample. The additional implementation of remote treatment for eating disorders will further advance access to care for those in need and only serve to improve overall patient outcomes.

There are limitations to this study. First, our sample did not include a comparison group; hence, we are unable to draw definite conclusions about the effectiveness of CBT-E delivered remotely compared with an in-person setting. However, the primary question addressed by this study is not whether CBT-E is effective but whether CBT-E retains clinical utility when delivered within the context of our intentionally remote treatment program. In this context, a within-person pre-post design is appropriate. Second, the sample was somewhat homogeneous due to self-selection in treatment, yet it was more diverse than most clinical trials [39,40,44,46]. Relatedly, we evaluated a patient sample from a single treatment program, so it is possible that results may not generalize outside of this organization. Future multisite studies should be conducted with an in-person comparison group so that more general and definitive claims about effectiveness can be made.

Third, as we noted earlier, the variability of time in treatment makes comparisons to clinical trials challenging; however, this is the reality of real-world treatment where patients leave treatment for a variety of reasons, including financial, ambivalence toward recovery, or other. Moreover, the variability in treatment length for our patients makes it challenging to evaluate a clear end-of-treatment remission and recovery rate as an outcome. Finally, we also noted that missing data yielded different analytic samples across our analyses. However, we found that imputing the missing data led to nearly identical results.

### Conclusions

This study provides real-world evidence that CBT-E, when intentionally adapted for remote delivery and implemented by a multidisciplinary care team, is associated with significant clinical improvements for adult patients with an eating disorder. By week 40 of treatment, the probability of reaching 95% of one's target weight was 0.50, and the probability of reaching subclinical levels of eating disorder, depression, and anxiety symptoms ranged from 0.48 to 0.56; outcomes that are consistent with those reported in clinical trials of in-person treatment. While the absence of a comparison group and reliance on a single treatment setting limit causal inference and generalizability, these results underscore the potential of remote CBT-E as an effective and scalable treatment option in outpatient settings. Future research should build on these findings, with multicenter studies incorporating an in-person delivery comparison group and exploring predictors of treatment response to inform individualized care.

## Appendix

Multimedia Appendix 1 Supplementary tables presenting patient characteristics by diagnosis (Tables S1 and S2) and model estimates overall (Table S3) and by diagnosis (Table S4).

## Abbreviations

AN: anorexia nervosa
BED: binge eating disorder
BN: bulimia nervosa
CBT: cognitive behavioral therapy
CBT-E: enhanced cognitive behavioral therapy
DSM: Diagnostic and Statistical Manual of Mental Disorders
EDE-Q: Eating Disorder Examination Questionnaire
GAD-7: Generalized Anxiety Disorder-7
HIPAA: Health Insurance Portability and Accountability Act
OSFED: other specified feeding or eating disorder
PHQ-8: Patient Health Questionnaire-8

## Data Availability

The datasets generated or analyzed during this study are not publicly available as they are derived from patient medical records and, therefore, protected health information. Deidentified data may be made are available from the corresponding author upon reasonable request.
