# Supplementary material for: Remotely Delivered Cognitive Behavioral Therapy for Adults With an Eating Disorder: Retrospective Analysis of a Real-World Patient Sample
Source: J Med Internet Res. 2025 Sep 18;27:e76464. doi: 10.2196/76464 (PMC12491894; doi:10.2196/76464)
Supplement: Multimedia Appendix 1 [file jmir_v27i1e76464_app1.docx]

| **Table S1.** Patient characteristics by diagnosis. | | | | |
| --- | --- | --- | --- | --- |
| **Variable** | **AN** | **BED** | **BN** | **OSFED** |
|  |  |  |  |  |
|  | *M* (*SD*) | *M* (*SD*) | *M* (*SD*) | *M* (*SD*) |
| Age | 27.06 (10.45) | 38.43 (12.06) | 30.85 (11.87) | 30.77 (11.07) |
|  | n (%) | n(%) | n(%) | n(%) |
| *Gender* |  |  |  |  |
| Cis-gender female | 790 (56.3) | 323 (23) | 108 (7.7) | 182 (13) |
| Cis-gender male | 58 (45.3) | 51 (39.8) | 9 (7) | 10 (7.8) |
| Transgender/Non-binary/Other | 56 (65.1) | 14 (16.3) | 1 (1.2) | 15 (17.4) |
| *Missing gender* | 9 (75) |  | 2 (16.7) | 1 (8.3) |
| *Race/Ethnicity* |  |  |  |  |
| White | 682 (58.5) | 265 (22.7) | 77 (6.6) | 142 (12.2) |
| Multi-ethnic/racial | 89 (53.6) | 37 (22.3) | 18 (10.8) | 22 (13.3) |
| Asian | 51 (60.7) | 13 (15.5) | 6 (7.1) | 14 (16.7) |
| Hispanic | 45 (47.9) | 30 (31.9) | 8 (8.5) | 11 (11.7) |
| Black/African American | 27 (40.9) | 23 (34.8) | 4 (6.1) | 12 (18.2) |
| Other | 11 (39.3) | 9 (32.1) | 3 (10.7) | 5 (17.9) |
| *Missing ethnicity* | 8 (32) | 11 (44) | 4 (16) | 2 (8) |
| *Prior Treatment* |  |  |  |  |
| No prior tx | 387 (79.47) | 21 (4.31) | 30 (6.16) | 49 (10.06) |
| Other prior tx | 370 (42.24) | 307 (35.05) | 74 (8.45) | 125 (14.27) |
| Prior HLOC | 118 (59.3) | 43 (21.61) | 12 (6.03) | 26 (13.07) |
| Unknown | 38 (56.72) | 17 (25.37) | 4 (5.97) | 8 (11.94) |

| **Table S2.** Patient starting BMI by diagnosis. | | | | | | | |
| --- | --- | --- | --- | --- | --- | --- | --- |
| **Diagnosis** | **M** | **SD** | **Min** | **25th Percentile** | **Median** | **75th Percentile** | **Max** |
| AN | 21.71 | 6.38 | 12.07 | 17.94 | 20.08 | 23.29 | 64.14 |
| BED | 38.39 | 10.15 | 15.16 | 31.31 | 37.42 | 43.92 | 72.62 |
| BN | 29.29 | 9.23 | 17.47 | 22.76 | 26.57 | 33.81 | 62.9 |
| OSFED | 30.56 | 9.65 | 14.81 | 23.38 | 28.31 | 36.02 | 72.08 |

| **Table S3.** Standardized multi-level model results for each outcome. Sample n varies across analyses due to missing data on key variables. | | | | | | | | | | | | | | |
| --- | --- | --- | --- | --- | --- | --- | --- | --- | --- | --- | --- | --- | --- | --- |
|  | **EDE-Q**  (n_obs_ = 4120; n_patients_ = 1410) | | | |  | **Anxiety**  (n_obs_ = 4476; n_patients_ = 1462) | | | |  | **Depression**  (n_obs_ = 4458; n_patients_ = 1465) | | | |
| **Variable** | ***b* (*se*)** | **Lower**  **CI** | **Upper**  **CI** | ***P*** |  | ***b* (*se*)** | **Lower**  **CI** | **Upper**  **CI** | ***P*** |  | ***b* (*se*)** | **Lower**  **CI** | **Upper**  **CI** | ***P*** |
| **Fixed effects:** |  |  |  |  |  |  |  |  |  |  |  |  |  |  |
| Intercept | 0.46 (0.04) | 0.38 | 0.54 | <.001 |  | 0.42 (0.04) | 0.34 | 0.51 | <.001 |  | 0.29 (0.04) | 0.2 | 0.38 | <.001 |
| Age | <0.01 (<0.01) | <0.01 | <0.011 | .061 |  | -0.01 (0) | -0.01 | <0.01 | .001 |  | 0 (0) | -0.01 | <0.01 | .049 |
| *Log*(Treatment week) | -0.27 (0.02) | -0.3 | -0.24 | <.001 |  | -0.15 (0.01) | -0.18 | -0.12 | <.001 |  | -0.14 (0.02) | -0.18 | -0.11 | <.001 |
| *Gender (Cis-gender female = 0)* |  |  |  |  |  |  |  |  |  |  |  |  |  |  |
| Cis-gender male | -0.2 (0.1) | -0.4 | -0.01 | .003 |  | -0.3 (0.1) | -0.5 | -0.09 | <.001 |  | -0.17 (0.1) | -0.37 | 0.03 | .009 |
| Transgender/Non-binary/Other | 0.3 (0.11) | 0.08 | 0.52 | <.001 |  | 0.27 (0.12) | 0.03 | 0.5 | .002 |  | 0.47 (0.12) | 0.24 | 0.71 | <.001 |
| *Diagnosis (AN = 0)* |  |  |  |  |  |  |  |  |  |  |  |  |  |  |
| BED | 0.07 (0.07) | -0.06 | 0.2 | .027 |  | -0.26 (0.07) | -0.4 | -0.12 | <.001 |  | 0.04 (0.07) | -0.1 | 0.17 | .06 |
| BN | 0.21 (0.1) | 0.01 | 0.4 | .004 |  | 0.06 (0.11) | -0.15 | 0.27 | .058 |  | 0.15 (0.11) | -0.06 | 0.36 | .016 |
| OSFED | 0.12 (0.08) | -0.03 | 0.27 | .011 |  | 0.01 (0.08) | -0.15 | 0.17 | .087 |  | 0.21 (0.08) | 0.05 | 0.36 | .001 |
| Age x Treatment week | <0.01 (<0.01) | <0.01 | <0.010 | .001 |  | <0.01 (<0.01) | <0.01 | <0.01 | .013 |  | <0.01 (<0.01) | <0.01 | <0.01 | .022 |
| Cis-gender male x Treatment week | -0.05 (0.04) | -0.12 | 0.02 | .016 |  | 0.01 (0.03) | -0.06 | 0.07 | .087 |  | 0 (0.04) | -0.07 | 0.07 | .091 |
| Transgender/Non-binary/Other x Treatment week | -0.01 (0.04) | -0.09 | 0.07 | .075 |  | -0.02 (0.04) | -0.09 | 0.06 | .069 |  | -0.01 (0.04) | -0.09 | 0.06 | .071 |
| BED x Treatment week | -0.06 (0.02) | -0.1 | -0.01 | .002 |  | -0.01 (0.02) | -0.05 | 0.04 | .082 |  | -0.08 (0.02) | -0.13 | -0.04 | <.001 |
| BN x Treatment week | -0.07 (0.04) | -0.14 | 0.01 | .008 |  | -0.01 (0.04) | -0.08 | 0.06 | .071 |  | -0.08 (0.04) | -0.15 | <0.01 | .003 |
| OSFED x Treatment week | -0.01 (0.03) | -0.06 | 0.05 | .083 |  | 0.01 (0.02) | -0.04 | 0.06 | .069 |  | -0.02 (0.03) | -0.08 | 0.03 | .034 |
| **Random effects:** |  |  |  |  |  |  |  |  |  |  |  |  |  |  |
| SD (Patient) | 0.8 |  |  |  |  | 0.84 |  |  |  |  | 0.84 |  |  |  |
| SD (Treatment Week) | 0.21 |  |  |  |  | 0.16 |  |  |  |  | 0.19 |  |  |  |

| **Table S4. Weekly estimates for each outcome by diagnosis** | | | | | | | |
| --- | --- | --- | --- | --- | --- | --- | --- |
| **Outcome** | **Diagnosis** | **Week 0** | **Week 4** | **Week 8** | **Week 20** | **Week 40** | **Week 52** |
| ED symptoms | AN | 3.697 [CI: 3.537, 3.856] | 3.076 [CI: 2.927, 3.226] | 2.85 [CI: 2.69, 3.01] | 2.523 [CI: 2.337, 2.709] | 2.265 [CI: 2.053, 2.478] | 2.166 [CI: 1.943, 2.39] |
|  | BED | 3.803 [CI: 3.614, 3.993] | 3.05 [CI: 2.871, 3.229] | 2.775 [CI: 2.583, 2.967] | 2.379 [CI: 2.155, 2.602] | 2.066 [CI: 1.811, 2.321] | 1.945 [CI: 1.677, 2.214] |
|  | BN | 4.002 [CI: 3.698, 4.306] | 3.227 [CI: 2.945, 3.508] | 2.943 [CI: 2.642, 3.245] | 2.535 [CI: 2.184, 2.886] | 2.213 [CI: 1.81, 2.615] | 2.089 [CI: 1.665, 2.513] |
|  | OSFED | 3.877 [CI: 3.642, 4.112] | 3.243 [CI: 3.028, 3.458] | 3.011 [CI: 2.784, 3.239] | 2.678 [CI: 2.417, 2.939] | 2.414 [CI: 2.118, 2.711] | 2.313 [CI: 2.002, 2.625] |
| Depression | AN | 12.17 [CI: 11.49, 12.85] | 10.873 [CI: 10.279, 11.468] | 10.4 [CI: 9.782, 11.018] | 9.717 [CI: 9.018, 10.416] | 9.178 [CI: 8.387, 9.969] | 8.971 [CI: 8.14, 9.803] |
|  | BED | 12.385 [CI: 11.569, 13.201] | 10.3 [CI: 9.583, 11.017] | 9.538 [CI: 8.791, 10.286] | 8.441 [CI: 7.593, 9.288] | 7.574 [CI: 6.614, 8.534] | 7.241 [CI: 6.232, 8.251] |
|  | BN | 13.049 [CI: 11.748, 14.351] | 11 [CI: 9.87, 12.131] | 10.252 [CI: 9.071, 11.433] | 9.173 [CI: 7.825, 10.521] | 8.321 [CI: 6.784, 9.858] | 7.994 [CI: 6.375, 9.613] |
|  | OSFED | 13.397 [CI: 12.414, 14.379] | 11.865 [CI: 11.016, 12.714] | 11.306 [CI: 10.433, 12.178] | 10.499 [CI: 9.53, 11.468] | 9.862 [CI: 8.776, 10.949] | 9.618 [CI: 8.48, 10.757] |
| Anxiety | AN | 11.635 [CI: 10.972, 12.297] | 10.359 [CI: 9.78, 10.938] | 9.893 [CI: 9.296, 10.49] | 9.222 [CI: 8.557, 9.887] | 8.692 [CI: 7.946, 9.437] | 8.488 [CI: 7.707, 9.27] |
|  | BED | 10.15 [CI: 9.355, 10.945] | 8.827 [CI: 8.129, 9.524] | 8.343 [CI: 7.623, 9.064] | 7.647 [CI: 6.842, 8.451] | 7.097 [CI: 6.193, 8] | 6.885 [CI: 5.939, 7.832] |
|  | BN | 11.979 [CI: 10.703, 13.255] | 10.58 [CI: 9.473, 11.687] | 10.07 [CI: 8.926, 11.213] | 9.333 [CI: 8.053, 10.614] | 8.752 [CI: 7.309, 10.195] | 8.529 [CI: 7.014, 10.043] |
|  | OSFED | 11.709 [CI: 10.75, 12.668] | 10.523 [CI: 9.695, 11.352] | 10.09 [CI: 9.246, 10.934] | 9.466 [CI: 8.541, 10.39] | 8.973 [CI: 7.946, 9.999] | 8.784 [CI: 7.711, 9.857] |
| ED symptoms | Patient Avg. | 3.845 [CI: 3.689, 4.001] | 3.149 [CI: 3.004, 3.294] | 2.895 [CI: 2.74, 3.05] | 2.529 [CI: 2.349, 2.709] | 2.239 [CI: 2.034, 2.445] | 2.129 [CI: 1.912, 2.345] |
| Depression | Patient Avg. | 12.75 [CI: 12.088, 13.412] | 11.01 [CI: 10.434, 11.585] | 10.374 [CI: 9.776, 10.972] | 9.458 [CI: 8.781, 10.134] | 8.734 [CI: 7.967, 9.501] | 8.456 [CI: 7.65, 9.262] |
| Anxiety | Patient Avg. | 11.368 [CI: 10.722, 12.014] | 10.072 [CI: 9.511, 10.634] | 9.599 [CI: 9.021, 10.177] | 8.917 [CI: 8.273, 9.561] | 8.378 [CI: 7.656, 9.101] | 8.171 [CI: 7.414, 8.929] |
